# DeSUMOylation of MKK7 kinase by the SUMO2/3 protease SENP3 potentiates lipopolysaccharide-induced inflammatory signaling in macrophages

**DOI:** 10.1074/jbc.M117.816769

**Published:** 2018-01-19

**Authors:** Yimin Lao, Kai Yang, Zhaojun Wang, Xueqing Sun, Qiang Zou, Xiaoyan Yu, Jinke Cheng, Xuemei Tong, Edward T. H. Yeh, Jie Yang, Jing Yi

**Affiliations:** From the ‡ Shanghai Key Laboratory of Tumor Microenvironment and Inflammation, Department of Biochemistry and Molecular Cell Biology, Institutes of Medical Sciences, Shanghai Jiao Tong University School of Medicine, Shanghai 200025, China,; the § Department of Immunology and Microbiology, Shanghai Jiao Tong University School of Medicine, Shanghai Institute of Immunology, Shanghai 200025, China, and; the ¶ Department of Internal Medicine, University of Missouri, Columbia, Missouri 65211

**Keywords:** c-Jun N-terminal kinase (JNK), inflammation, innate immunity, macrophage, sumoylation, ROS, SENP3

## Abstract

Protein SUMOylation has been reported to play a role in innate immune response, but the enzymes, substrates, and consequences of the specific inflammatory signaling events are largely unknown. Reactive oxygen species (ROS) are abundantly produced during macrophage activation and required for Toll-like receptor 4 (TLR4)–mediated inflammatory signaling. Previously, we demonstrated that SENP3 is a redox-sensitive SUMO2/3 protease. To explore any links between reversible SUMOylation and ROS-related inflammatory signaling in macrophage activation, we generated mice with *Senp3* conditional knock-out in myeloid cells. In bacterial lipopolysaccharide (LPS)-induced *in vitro* and *in vivo* inflammation models, we found that SENP3 deficiency markedly compromises the activation of TLR4 inflammatory signaling and the production of proinflammatory cytokines in macrophages exposed to LPS. Moreover, *Senp3* conditional knock-out mice were significantly less susceptible to septic shock. Of note, SENP3 deficiency was associated with impairment in JNK phosphorylation. We found that MKK7, which selectively phosphorylates JNK, is a SENP3 substrate and that SENP3-mediated deSUMOylation of MKK7 may favor its binding to JNK. Importantly, ROS-dependent SENP3 accumulation and MKK7 deSUMOylation rapidly occurred after LPS stimulation. In conclusion, our findings indicate that SENP3 potentiates LPS-induced TLR4 signaling via deSUMOylation of MKK7 leading to enhancement in JNK phosphorylation and the downstream events. Therefore this work provides novel mechanistic insights into redox regulation of innate immune responses.

## Introduction

The innate inflammatory responses in the human body must be precisely controlled to achieve a balance between pathogen clearance and tissue integrity. Therefore, inflammatory signaling in cells of the innate immune system is tightly regulated at multiple levels, including transcriptionally, post-transcriptionally, translationally, and post-translationally (1).

Toll-like receptors (TLRs)^3^ sense a wide range of invading pathogens, including bacteria, fungi, and viruses, as well as their components in antigen-presenting cells. Activation of several signaling pathways downstream of TLRs, such as NF-κB, activator protein-1 (AP-1), and interferon regulatory factors, mediates the formation of new gene expression profiles in response to pathogens and their components (2–7). Given that post-translational modifications (PTMs) are a type of rapid modulation, how PTMs are induced by pathogens and pathogenic compounds during the inflammatory responses and consequently regulate TLR signaling have been paid intensive attention (1). The classical and most widely known PTMs are phosphorylation and ubiquitination, such as the kinase phosphorylation cascade in the mitogen-activated protein kinase (MAPK)/AP-1 pathway and IκB ubiquitination in the NFκB pathway (8–10). Engagement of so-called “unconventional” PTMs in innate immune responses is an emerging research field that mainly focuses on methylation, acetylation, and SUMOylation (1, 11).

SUMOylation is a reversible PTM in which substrate proteins are conjugated with SUMO1 or SUMO2/3 proteins through an E1, E2, and E3 cascade and are deconjugated by members of the SENP family (12, 13). SUMOylation controls protein stability, localization, activity, and interaction, thus affecting cell signaling and gene expression in a variety of tissues (14, 15). During the past decade, a great body of research has identified SUMOylation as an important regulator in antiviral signaling in both positive and negative manners (16–28). In contrast, SUMOylation in immune responses to bacteria and their compounds has not been adequately studied (18, 20, 29–31). Recently, two papers have described the roles of the SUMO E2-conjugating enzyme UBC9 in Kupffer cells, bone marrow-derived dendritic cells, macrophages, and the RAW264.7 cell line in response to either virus or bacterial lipopolysaccharide (LPS), and have reported negative regulation of inflammation by global SUMOylation (20, 29). Thus, the regulatory effects of SUMOylation vary depending on the types of pathogens, stimuli, and innate immune cells. To better understand the role of SUMOylation in the innate immune response, it is necessary to characterize the specific enzymes involved in SUMO conjugation/deconjugation balance as well as their key substrates under specific inflammatory conditions.

We have previously found that the SUMO protease SENP3, which is specifically responsible for the removal of SUMO2/3 from substrates, is a redox-sensitive enzyme (32, 33). Among the SENP family members, SENP3 is unique in its rapid increases in protein level, because the abrogation of ubiquitin-mediated degradation after an oxidation following a mild increase in reactive oxygen species (ROS). Various oxidative stress conditions have been found to induce SENP3 stabilization, including exposing cells to the most important ROS, hydrogen peroxide (H_2_O_2_), chemical hypoxia, the inflammation-causing and carcinogenic cigarette compound, the cytokine interleukin 6, or a cancerous environment (32–37).

Macrophages produce high levels of ROS during their contact with invading pathogens, pathogenic components, and other immune cells, which are required for both pathogen clearance and inflammatory signaling (38–41). We thus presumed that SENP3 might play a role in the macrophage-mediated host innate immune response.

To test this hypothesis, we constructed *Senp3*^+/−^ and *Senp3^flox/flox^ Lyz2-cre* mice (mice with SENP3 conditional knock-out in myeloid cells, named *Senp3* cKO mice for short) to investigate the roles of SENP3 and SUMO2/3 modifications in ROS-related inflammatory signaling in macrophages. We used the murine macrophage cell line RAW264.7 (RAW cells) with SENP3 expression knocked down by small interfering RNA (siRNA) and primary bone marrow-derived macrophages (BMDM) from *Senp3*^+/−^ or cKO mice. We found that SENP3 potentiates TLR4 inflammatory signaling in macrophages exposed to LPS. SENP3 deficiency compromised inflammatory cytokine production in macrophages, which was correlated with a selective impairment of c-Jun N-terminal kinase (JNK) phosphorylation after LPS stimulation. We verified the upstream kinase MKK7, which selectively phosphorylates JNK but not p38, as a substrate of SENP3; deSUMOylation of MKK7 might favor MKK binding to JNK. Importantly, SENP3 was rapidly accumulated in various types of macrophages after LPS stimulation in a ROS-dependent manner, and SENP3-mediated MKK7 deSUMOylation occurred in BMDM under LPS stimulation. Finally, we demonstrated that *Senp3* cKO mice, compared with their wild-type counterparts, exhibited lower cytokine levels in serum and organs, as well as longer survival in LPS-induced endotoxin shock. Therefore, this study verifies that SENP3 potentiates LPS-induced TLR4 signaling via deSUMOylation of MKK7, which dissects a link between SUMOylation and ROS-related inflammatory signaling in macrophage activation.

## Results

### SENP3 deficiency decreases LPS-induced cytokine production in macrophages

**Figure 1.**
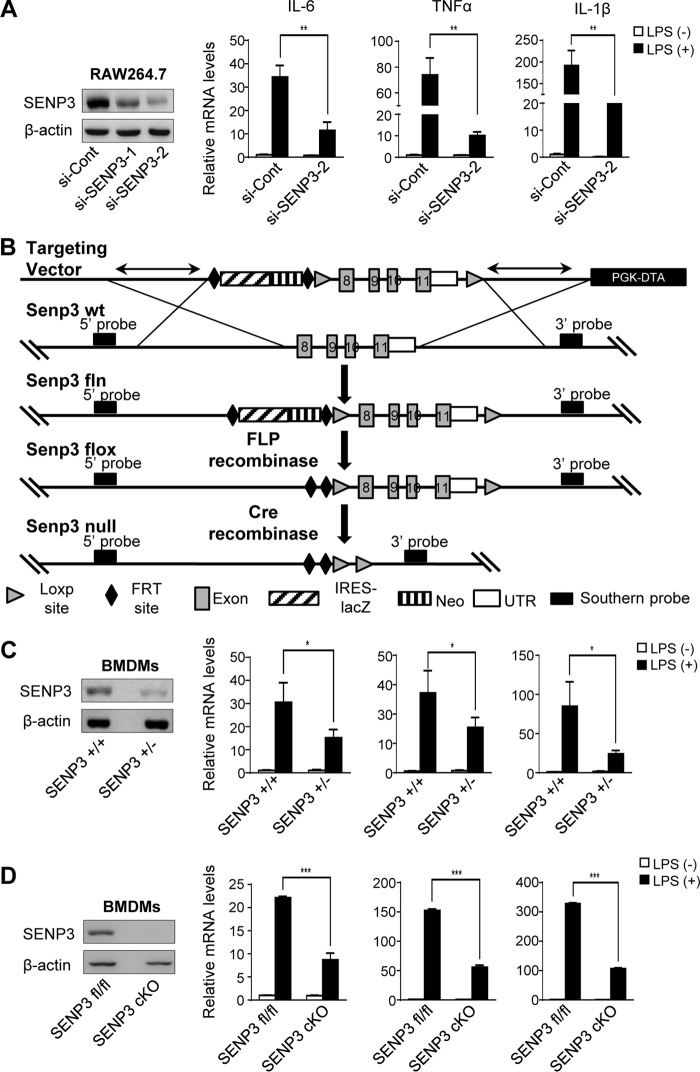
**SENP3 deficiency decreases LPS-induced cytokine production in macrophages.** *A,* RAW 264.7 cells transfected with nonspecific siRNA (*si-Cont*) or SENP3 siRNA (*si-SENP3*) were incubated with LPS (100 ng/ml) for 6 h. mRNA levels of proinflammatory cytokines IL-6, TNFα, and IL-1β were assessed by qRT-PCR. The knockdown efficiency of two siRNAs of SENP3 was determined by IB. *B,* a strategy of *Senp3^flox/flox^ Lyz2-cre* mouse generation was shown. A mouse model expressing a myeloid cell-specific deletion of SENP3 was generated using transgenic mice bearing loxp sites flanking exon 8 to exon 11 of the *Senp3* gene (*SENP3^flox^*) and mice expressing a Cre recombinase transgene from the Lysozyme M locus (Lys M-Cre also known as Lyz2-Cre). *C,* BMDMs isolated from *Senp3*^+/+^ and *Senp3*^+/−^ mice were incubated with LPS (100 ng/ml) for 6 h. mRNA levels of proinflammatory cytokines IL-6, TNFα, and IL-1β were assessed by qRT-PCR. The SENP3 level was determined by IB. *D,* BMDMs isolated from *Senp3^flox/flox^* (*Senp3^fl/fl^*) and *Senp3^flox/flox^ Lyz2-cre* (*Senp3 cKO*) mice were incubated with LPS (100 ng/ml) for 6 h. mRNA levels of proinflammatory cytokines IL-6, TNFα, and IL-1β were assessed by qRT-PCR. SENP3 level was determined by IB. Graphs show the mean ± S.D. and data (*A, C*, and *D*) shown are representative of three independent experiments. *, *p* < 0.05; **, *p* < 0.01; ***, *p* < 0.001.

We examined the expression of the major inflammatory cytokines in macrophages exposed to 100 ng/ml LPS for 6 h. The expression of SENP3 was knocked down using siRNA in the murine macrophage RAW cells. The results of quantitative reverse transcription PCR showed that the mRNA transcriptional levels of IL-6, TNFα, and IL-1β were significantly lower in SENP3 knock-down (si-SENP3) cells compared with nonspecific siRNA control (si-con) cells (Fig. 1*A*). We then determined the correlations between SENP3 levels and cytokine transcription in BMDM of *Senp3*-knock-out mice. Because *Senp3*^−/−^ mice were embryonic lethal,^4^ *Senp3*^+/−^ mice were generated, and their genotype was validated (Fig. S1*A*). Furthermore, we designed a vector to specifically knock-out SENP3 expression in myeloid cells to generate *Senp3^flox/flox^ Lyz2-cre* mice (Fig. 1*B*), and the genotype of these cKO mice was validated (Fig. S1*B*). The BMDM, induced from hematopoietic precursor cells *in vitro*, were analyzed by flow cytometry; the purity was above 95% (Fig. S1*C*). The SENP3 expression levels in the BMDM of these mice were verified. In the BMDM of *Senp3*^+/−^ mice and *Senp3^flox/flox^ Lyz2-cre* mice, similarly to RAW cells, SENP3 deficiency impaired the mRNA induction of IL-6, TNFα, and IL-1β by LPS to varying extents (Fig. 1, *C* and *D*). Therefore, SENP3 may play a role in LPS-induced cytokine production in macrophages.

### SENP3 deficiency selectively attenuates MAPK signaling and JNK phosphorylation in macrophages

**Figure 2.**
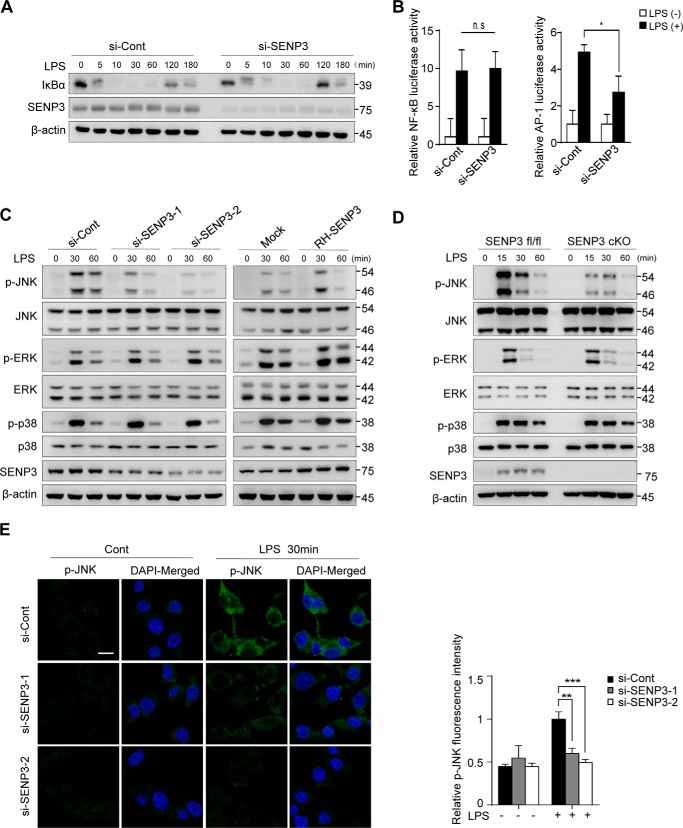
**SENP3 deficiency selectively attenuates MAPK signaling and JNK phosphorylation in macrophages.** *A,* RAW 264.7 cells transfected with si-Cont or si-SENP3 were stimulated with LPS (100 ng/ml) for the indicated time. IκBα degradation was assessed by IB. *B,* NF-κB-luciferase (*left panel*) or AP-1-luciferase (*right panel*) and *Renilla* were transfected into RAW 264.7 cells together with the indicated siRNA. 48 h after transfection, cells were stimulated with LPS (100 ng/ml) for 6 h followed by luciferase reporter assays. Graphs show the mean ± S.D. and data shown are representative of three independent experiments. *ns,* no statistical difference; *, *p* < 0.05. *C,* RAW 264.7 cells transfected with si-Cont or si-SENP3 were stimulated with LPS (100 ng/ml) for the indicated time (*left panel*). The cells were overexpressed with RGS-His-SENP3 (*RH-SENP3* for short) (*right panel*). Phosphorylated JNK (p-JNK), p38 (*p-p38*), and ERK (*p-ERK*) were assessed by IB. *D, Senp3^fl/fl^* and *Senp3* cKO BMDMs stimulated with LPS (100 ng/ml) for the indicated time. p-JNK, p-p38, and p-ERK were assessed by IB. *E,* RAW 264.7 cells were stimulated with LPS (100 ng/ml) for 30 min. Immunofluorescence of p-JNK was performed and representative pictures were shown in *left panel*. Quantification of p-JNK fluorescence intensity was shown in the *right panel*. p-JNK, *green*; 4′,6-diamidino-2-phenylindole (DAPI), *blue. Scale bar*, 10 μm. Graphs are shown as mean ± S.D. (*n* = 40).

TLR4 signaling triggered by LPS mainly activates the transcriptional activity of NF-κB and AP-1, which lead to the transcription of distinct cytokine genes. We first examined which of these two signaling pathways SENP3 might affect. The patterns of IκB degradation remained almost the same between si-SENP3 and si-con RAW cells (Fig. 2*A*). Luciferase reporter assay showed that the transcriptional activity of NF-κB was not affected but that of AP-1 was inhibited by SENP3 knockdown in RAW cells (Fig. 2*B*). These data indicated a selective role of SENP3 in the regulation of the MAPK signaling pathway.

There are three major groups of MAPKs in macrophages that mediate inflammatory signaling downstream of TLR4: extracellular signal-regulated protein kinases (ERK), p38 MAP kinases, and c-Jun NH_2_-terminal kinases (JNK1/2). We detected the time courses of phosphorylation of ERK, p38, and JNK after rapid stimulation of LPS in RAW cells with SENP3 knockdown or overexpression. The results of immunoblotting showed that all three MAPKs were activated during the first 15 min by LPS. However, phosphorylation of JNK was significantly decreased by SENP3 knockdown and was increased by SENP3 overexpression (Fig. 2*C* and Fig. S2, *A–C*); in contrast, the phosphorylation levels of ERK and p38 were not altered under either condition (Fig. 2*C*). Moreover, the same phenomenon of decreased JNK phosphorylation was observed in the BMDM of *Senp3* cKO mice (Fig. 2*D* and Fig. S2*D*) as well as *Senp3*^+/−^ mice (Fig. S2, *E* and *F*). Immunofluorescence staining for phosphorylated JNK in RAW cells also demonstrated markedly decreased JNK activation in SENP3 knockdown cells after LPS stimulation (Fig. 2*E*). Together, these data indicated that SENP3 enhances macrophage JNK activation in response to LPS.

### SENP3 catalyzes deSUMOylation of MKK7 at the Lys-18 site

**Figure 3.**
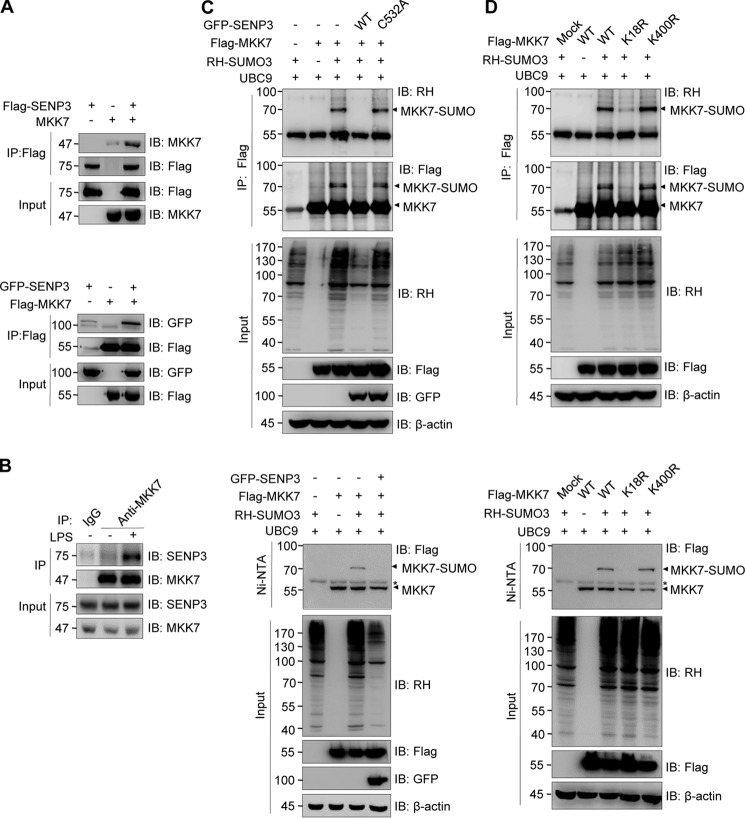
**SENP3 catalyzes deSUMOylation of MKK7 at Lys-18 site.** *A,* the interaction of SENP3 with MKK7 was determined by co-IP. HEK293T cells were transfected with FLAG–SENP3 and -MKK7 plasmids for 48 h. Co-IP was performed using FLAG-M2 beads for immunoprecipitation and using anti-MKK7 antibodies for IB (*upper panel*). HEK293T cells were transfected with GFP–SENP3 and FLAG–MKK7 plasmids for 48 h. Co-IP was performed using FLAG-M2 beads for immunoprecipitation and using anti-GFP antibodies for IB (*bottom panel*). *B,* TLR4 293 cells were administrated with LPS (100 ng/ml) for 30 min. The interaction of endogenous SENP3 and MKK7 was determined by co-IP. *C,* SUMO3 conjugates of MKK7 were determined by denaturing co-IP or Ni-bead pull-down. FLAG–MKK7 were transfected into HEK293T cells along with RH–SUMO3, UBC9, and GFP–SENP3 or GFP–SENP3 mutant (*C532A*) as indicated for 48 h. Cell lysates were immunoprecipitated with FLAG-M2 beads, and then analyzed by IB with the indicated antibodies (*upper panel*). FLAG–MKK7 were transfected into HEK293T cells along with RH-SUMO3, UBC9, and GFP–SENP3 as indicated for 48 h. Cells were lysed and RH–SUMO3 was pulled down using Ni-NTA beads and then analyzed by IB as indicated (*bottom panel*). *D,* SUMO3 conjugates of MKK7 were determined by denaturing co-IP or Ni-bead pull-down. HEK293T cells were transfected with FLAG-tagged MKK7 WT or mutated K18R and K400R, RH–SUMO3 and UBC9 as indicated for 48 h. Cell lysates were immunoprecipitated with FLAG-M2 beads, and then analyzed by IB with the indicated antibodies (*upper panel*). Cells were lysed and RH–SUMO3 was pulled down using Ni-NTA beads and then analyzed by IB as indicated (*bottom panel*). *Arrowheads* indicated SUMO3-conjugated MKK7 in (*C* and *D*). *, nonspecific bands.

SENP3 is a SUMO protease that removes the SUMO2/3 modification from substrate proteins and affects their activity and function. Naturally, the effect of SENP3-mediated enhancement in JNK phosphorylation might depend on its enzymatic activity in de-conjugating SUMO2/3 from a protein in the JNK signaling pathway. MKK4/7 are MAPKKs upstream of both JNK and p38. Because the phosphorylation of p38 and ERK was not altered by SENP3, the MKK specific for JNK but not p38, *i.e.* MKK7, was first considered as the potential substrate of SENP3. As predicted by freely available software, MKK7 had a high probability of SUMOylation and Lys-18 and Lys-400 were highly scored SUMOylation sites (Fig. S3). We first tested whether SENP3 interacted with MKK7 in a HEK293T cell overexpression system. Co-immunoprecipitation (IP) assays using antibodies against the tagged SENP3, MKK7, or endogenous MKK7 were performed, in both directions. The results showed that there was an interaction between SENP3 and MKK7 (Fig. 3*A*). TLR4/MD2 HEK293 cells, *i.e.* HEK293 cells with stable overexpression of TLR4 and MD2 (named TLR4 293 in the subsequent text), are frequently used as a cell line for investigating macrophage inflammatory signaling events under ectopic expression conditions (42–44). The interaction of endogenous SENP3 and MKK7 was assessed in TLR4 293 cells by a co-IP assay. The results of co-IP showed that SENP3 and MKK7 interacted, and this interaction was enhanced when TLR4 293 cells were exposed to LPS (Fig. 3*B*).

We next tested whether MKK7 had a SUMO2/3 modification and could be deSUMOylated by SENP3 in HEK293T cells. SUMOylation of MKK7 was examined by using FLAG-bead IP and Ni-bead pull-down assays in SUMO3 overexpression cells. SUMO3 conjugation of MKK7 displayed a prominent band on the gel at a 75-kDa molecular mass in both assays, thus indicating that one SUMO conjugate was bound to MKK7 (∼55 kDa in molecular mass) (Fig. 3*C*). Wild-type SENP3 (WT) was able to remove SUMO3 from MKK7 (Fig. 3*C*), whereas the SENP3 mutant (C532A, lacking enzymatic activity) was not able to do so (Fig. 3*C, upper panel*). To map the SUMOylation site of MKK7, we constructed plasmids for expression of mutants in which the predicted Lys residues of 18 and 400 were replaced by Arg, thus preventing SUMOylation. SUMOylation of MKK7 was again examined using FLAG IP and Ni-bead pull-down assays. The results clearly showed that MKK7 was conjugated with SUMO3 at Lys-18, because the WT MKK7 was pulled down with SUMO3 conjugates, displaying the SUMO band, and the K18R mutant lacked this band; however, the K400R mutant retained the SUMO band (Fig. 3*D*). These data verified MKK7 as the substrate of SENP3 and Lys-18 as the site of MKK SUMO3 modification.

### SENP3-mediated deSUMOylation of MKK7 potentiates JNK activation

**Figure 4.**
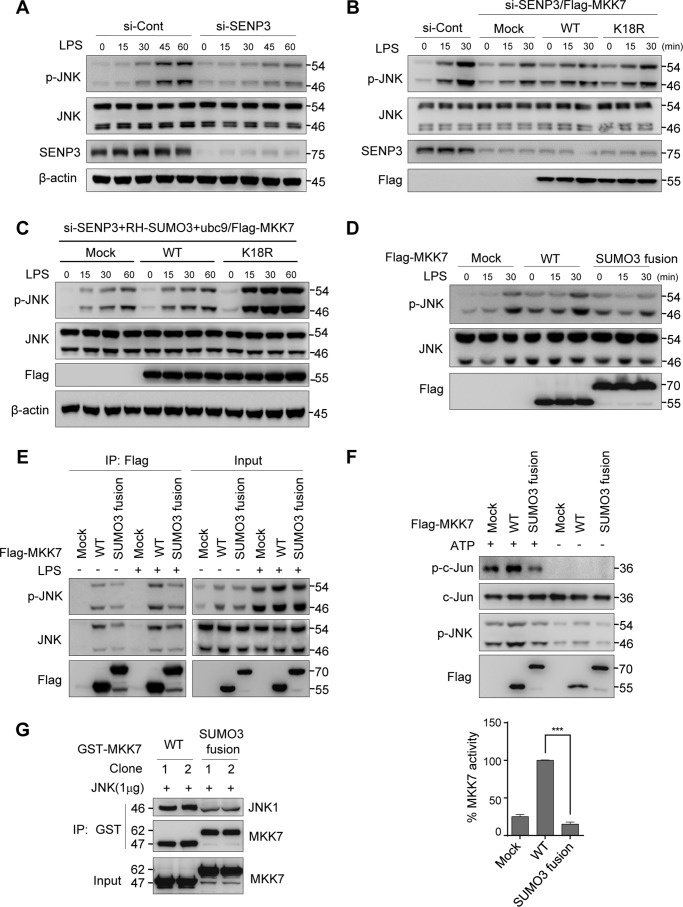
**SENP3-mediated deSUMOylation of MKK7 potentiates JNK activation.** *A,* TLR4 293 cells transfected with si-Cont or si-SENP3 for 48 h were incubated with LPS (100 ng/ml) for the indicated time, and p-JNK was assessed by IB. *B,* TLR4 293 cells with si-SENP3 were transfected with FLAG-tagged MKK7 WT or SUMO-less mutant K18R. The cells were incubated with LPS (100 ng/ml) for the indicated time and then p-JNK was determined by IB. *C,* TLR4 293 cells with si-SENP3 were co-transfected with RH–SUMO3, UBC9, and FLAG-tagged MKK7 WT or SUMO-less mutant K18R. The cells were incubated with LPS (100 ng/ml) for the indicated time and then p-JNK was determined by IB. *D,* TLR4 293 cells transfected with FLAG-tagged MKK7 WT or SUMO fusion plasmid for 48 h were stimulated with LPS (100 ng/ml) for the indicated time. p-JNK was analyzed by IB. *E,* TLR4 293 cells transfected with FLAG-tagged MKK7 WT or SUMO3 fusion for 48 h were stimulated with LPS (100 ng/ml) for 30 min. The interaction of MKK7 with p-JNK was determined by co-IP. *F*, TLR4 293 cells were transfected with FLAG-tagged MKK7 WT or SUMO3 fusion for 48 h. Immunoprecipitation kinase assay was performed. Phosphorylated JNK (*p-JNK*) and c-Jun (*p-c-Jun*) were harvested from anti-c-Jun-precipitated cell lysates. The levels of p-JNK and p-c-Jun were analyzed by IB and quantified in three independent experiments; graphs are shown as mean ± S.D. *G,* GST–MKK7 and GST–MKK7–SUMO3 were expressed in bacteria and purified. GST pull-down assays were used to test the binding capability between GST–MKK7 or GST–MKK7–SUMO3 and JNK1. JNK1 (1 μg) was tested for binding to GST–Sepharose with GST–MKK7 or GST–MKK7–SUMO3. JNK1 bound with MKK7 was determined by IB.

As TLR4 293 cells had endogenous SENP3–MKK7 interaction, we then examined whether a decrease of JNK phosphorylation correlated with SENP3 knockdown in these cells. Immunoblotting analysis demonstrated that despite that the peaks in LPS-induced phosphorylation of JNK appeared later in TLR4 293 cells than in RAW cells or BMDMs, the correlation was present in TLR4 293 cells as well; phosphorylation of JNK was decreased by SENP3 knockdown (Fig. 4*A*). TLR4 293 cells were thus suitable for use in further experiments. To test whether phosphorylation of JNK was regulated by deSUMOylation of MKK7 by SENP3, MKK7 WT and SUMO-less mutant K18R plasmids were expressed in si-SENP3 TLR4 293 cells. MKK7 mutant K18R led to higher JNK phosphorylation than WT did, which rescued the effects of si-SENP3 compared with si-con (Fig. 4*B*). When we overexpressed SUMO2/3 in these SENP3 knockdown cells, a more remarkable increase of JNK phosphorylation could be observed in K18R mutant-expressing cells, compared with that in WT-expressing cells (Fig. 4*C*). This might be attributed to that more SUMO2/3 conjugation to the WT sustained a difference in effects of a Lys-18–SUMO-less MKK7 over a SUMOylated one. Simultaneously, we constructed a fusion plasmid, MKK7–SUMO3 with a SUMO linked to the N terminus of MKK7, to mimic the existence of SUMO3 at the extreme N terminus of MKK7 (Lys-18). Overexpression of the MKK7–SUMO3 fusion protein in si-SENP3 TLR4 293 cells caused lower phosphorylation of JNK after LPS stimulation, compared with that of the WT (Fig. 4*D*).

Next, we elucidated how SUMOylation of MKK7 regulates the phosphorylation of JNK. MKK7 binds to JNK via its three conserved docking sites (D1, D2, and D3) in the N-terminal region, which contain amino acid residues (aa) 1 to 80 (45, 46). Notably, Lys-18 is located at docking site D1 (aa 1–38) of MKK7. Given that a Lys-18–SUMO-less MKK7 mutant increased JNK phosphorylation, we speculated that SUMO conjugation at this region might hinder the JNK binding moiety of MKK7. To test this hypothesis, an MKK7–SUMO3 fusion protein with a FLAG tag was overexpressed in TLR4 293 cells. A FLAG co-IP assay showed that MKK7–SUMO3 had weaker binding to both total JNK and phosphorylated JNK than WT MKK7. Moreover, after LPS stimulation, the interactions between MKK7 and p-JNK/JNK were increased, whereas MKK7–SUMO3 prevented this increase (Fig. 4*E*).

To further compare the kinase activity of the SUMO-fused MKK7 with its non-fused counterpart, we used a JNK Activity Screening Kit, in which MKK7–SUMO3 or MKK7-catalyzed products, *i.e.* phosphorylated JNK1 and its downstream c-Jun, were evaluated in an *ex vivo* reaction system with or without ATP. Only in ATP-plus reaction was the phosphorylated c-Jun detectable and the phosphorylated JNK1 was generated to some extent. But, MKK7–SUMO3 catalyzed much fewer products than MKK7, suggesting an inhibited kinase activity of MKK7–SUMO3 (Fig. 4*F*). An *in vitro* binding assay compared the binding capacity of GST–MKK7–SUMO3 and GST–MKK7 with a recombinant JNK, showing that MKK7–SUMO3 had weaker binding with JNK (Fig. 4*G*). In *in vitro* system GST–MKK7 was not SUMO conjugated, similar to a SUMO-less MKK7. Hence, these data suggested that deSUMOylation of MKK7 favors MKK binding to JNK, thus promoting JNK phosphorylation.

### SENP3 rapidly accumulates and deSUMOylates MKK7 after LPS stimulation

**Figure 5.**
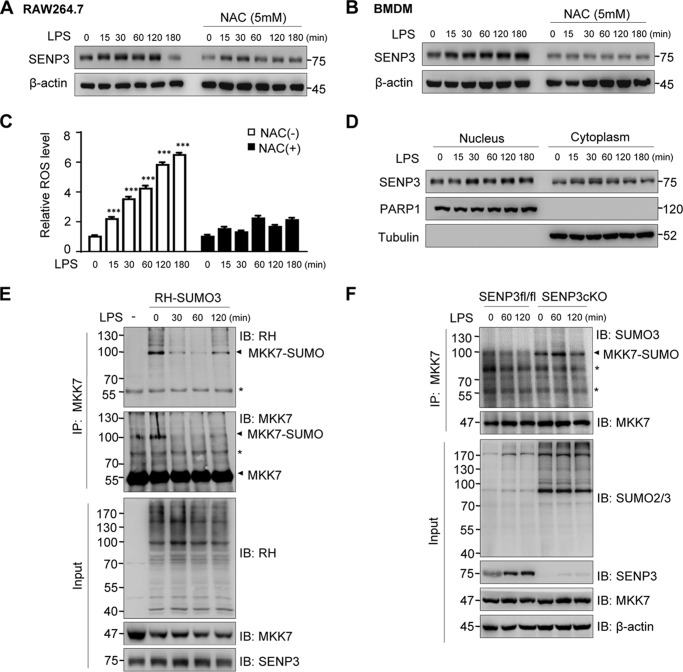
**SENP3 rapidly accumulates and deSUMOylates MKK7 after LPS stimulation.** *A,* RAW 264.7 cells were stimulated with LPS (100 ng/ml) in the presence or absence of 5 mm NAC for the indicated time. NAC was pretreated for 2 h. SENP3 accumulation was monitored by IB. *B,* BMDMs were stimulated with LPS (100 ng/ml) in the presence or absence of 5 mm NAC for the indicated time. SENP3 accumulation was monitored by IB. *C,* BMDMs were exposed to LPS (100 ng/ml) for the indicated time. The ROS level was determined by DCFH-DA staining and flow-cytometric analysis. NAC was pretreated for 2 h. Graphs show the mean ± S.D. of three independent experiments. ***, *p* < 0.001. *D,* RAW 264.7 cells were exposed to LPS (100 ng/ml) for the indicated time. SENP3 levels in cytoplasmic and nuclear fractions were evaluated by IB. Tubulin and poly(ADP-ribose) polymerase (*PARP*) were taken as the internal controls for cytoplasm and nucleus, respectively. To show SENP3 levels comparable between the cytoplasmic and nuclear fractions based on protein concentration measurements, the sample volume ratio of cytoplasmic fractions *versus* nuclear fractions was about 10:1. *E,* TLR4 293 cells were transfected with RH–SUMO3 for 48 h followed by stimulation with LPS (100 ng/ml) for the indicated time. Endogenous MKK7 that conjugated with RH–SUMO3 was detected by co-IP. *F, Senp3^fl/fl^* and *Senp3* cKO BMDMs were stimulated with LPS (100 ng/ml) for the indicated time. Endogenous MKK7 that conjugated with endogenous SUMO3 in the cells was detected by co-IP. *Arrowheads* indicated SUMO3-conjugated MKK7 in *E* and *F*; *, nonspecific bands.

The above data demonstrated the link between MKK7 SUMOylation and JNK phosphorylation as well as cytokine production in SENP3 knockdown or overexpression contexts. To demonstrate the regulatory role of SENP3 in LPS-induced inflammation, we sought to determine whether SENP3 indeed participated in the LPS-triggered events. We detected the SENP3 protein levels in RAW cells and BMDM exposed to LPS at a dose of 100 ng/ml. The SENP3 protein levels rapidly increased in whole RAW cell lysates in a pattern that started after 15 min and recovered after 2 h (Fig. 5*A*). Our previous studies have shown that the rapid increase in the SENP3 protein level results from a blockage of degradation because of cysteine oxidation, which can be blocked by antioxidants (33). Cells were then preincubated with the antioxidant *N*-acetyl-l-cysteine (NAC) before LPS treatment. This pre-treatment abolished LPS-induced SENP3 accumulation to a great extent (Fig. 5*A*). BMDM from naive C57BL/6 WT mice showed a similar response to LPS stimulation, but the induction of SENP3 lasted longer (Fig. 5*B*). Indeed, a time-dependent increase in ROS generation was triggered by LPS, but this effect was blocked by pretreatment with NAC (Fig. 5*C*). Our previous studies have demonstrated that SENP3, a nucleolar protein, accumulates in the nucleoplasm under conditions of oxidative stress (32, 34). SENP3 has been reported to localize in the cytoplasm (47). To observe the cellular compartment where SENP3 localized after LPS induction, we examined the nuclear–cytoplasmic fractions of RAW cells. SENP3 was accumulated in both the nuclear and cytoplasmic fractions after LPS stimulation, but the accumulation occurred earlier in the cytoplasm (Fig. 5*D*). Collectively, these data demonstrated that SENP3 is up-regulated in macrophages in response to LPS, in a manner dependent on ROS.

We then assessed whether increased SENP3 catalyzed MKK deSUMOylation after LPS stimulation. TLR4 293 cells had a higher basal SENP3 level than those in RAW cells and BMDMs in the absence of LPS stimulation (Figs. 3*B* and 4, *A* and *B*, *versus* 5, *A* and *B*). To better visualize the changes in SUMOylation of MKK7 after LPS stimulation, we overexpressed RGS-His-tagged (Rh for short) SUMO3 in TLR4 293 cells and stimulated cells with LPS in a time course. The co-IP results showed that the SUMO3 conjugates of endogenous MKK7, as detected by antibodies against RH and MKK, respectively, were located at ∼100 kDa, probably indicative of two SUMO molecules bound to MKK7. SUMOylation of MKK7 was markedly decreased after LPS stimulation for 30 and 60 min, but recovered after 2 h of LPS treatment (Fig. 5*E*). Furthermore, BMDM of cKO mice were used, and immunoprecipitation of MKK7 was performed with an antibody against MKK7 before an antibody against SUMO2/3 was used to detect endogenous SUMO2/3 conjugates to endogenous MKK7. The results of the co-IP showed that SUMOylation of MKK7 was modest in SENP3 intact BMDM, and LPS-triggered deSUMOylation was scarcely discernible. However, the abundance of basal and LPS-triggered endogenous SUMO-conjugation of MKK7 appeared robustly in SENP3 knock-out BMDM, indicating a remarkable deSUMOylation otherwise occurred during LPS exposure (Fig. 5*F*). These results suggested that SENP3 potently deconjugates SUMO from MKK7 under LPS-induced inflammation scenarios.

### Senp3 cKO mice had less severe inflammatory responses and higher survival rates in LPS-induced endotoxin shock

**Figure 6.**
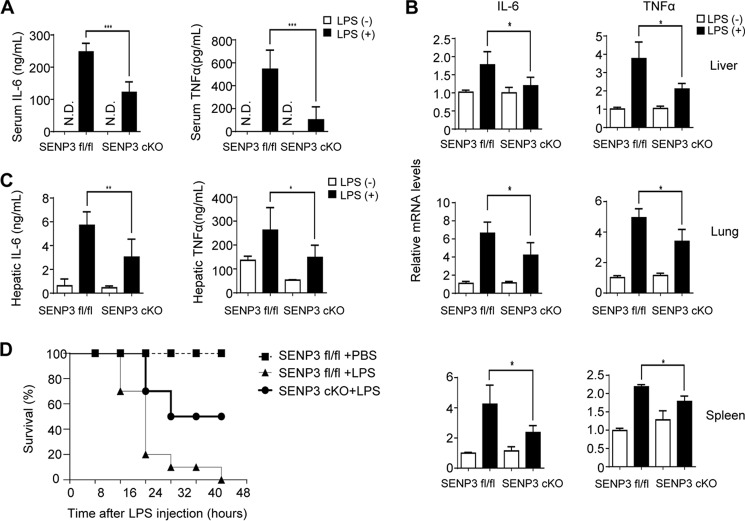
***Senp3* cKO mice had less severe inflammatory responses and higher survival rates in LPS-induced endotoxin shock.** *A–C*, *Senp3^fl/fl^* and *Senp3* cKO mice (*n* = 6–8 per group) were intraperitoneally injected with LPS (30 mg/kg) for 6 h. *A,* IL-6 and TNFα in mice serum were assessed by ELISA. *B,* TNFα and IL-6 expression in livers, lungs, and spleens (*n* = 6–8 per group) were monitored by qRT-PCR. Relative mRNA level of LPS injection group is to control group. *C,* IL-6 and TNFα in mice livers were assessed by ELISA. *D, Senp3^fl/fl^* and *Senp3* cKO mice (*n* = 10 per group) were injected with LPS (30 mg/kg, intraperitoneally) and then monitored for survival for up to 48 h. Graphs show the mean ± S.D. and data (*A–C*) are representative of three independent experiments. *, *p* < 0.05; **, *p* < 0.01; ***, *p* < 0.001.

To evaluate the systemic effects of SENP3 deficiency in macrophages and other leukocytes targeted by the *flox*/*flox Lyz2-cre* cKO strategy, we prepared an LPS endotoxin shock mouse model. The secretion levels of two major sepsis-related cytokines IL-6 and TNFα were measured by ELISA and showed a marked decrease in the sera of cKO mice compared with the WT mice (Fig. 6*A*). Their mRNA transcription levels were measured by qRT-PCR in tissue homogenates of livers, lungs, and spleens. The results showed that the IL-6 and TNFα mRNA levels were decreased to varying but statistically significant extents in three organs of cKO mice compared with the WT mice (Fig. 6*B*). The protein levels in the liver tissues of these cytokines were also decreased in cKO mice (Fig. 6*C*). Finally, the cKO mice showed much higher survival rates than the WT mice (Fig. 6*D*). Collectively, these data demonstrated that the cKO mice had generally less severe systemic inflammation.

## Discussion

In the present study, we reported a previously undescribed role of the SUMO protease SENP3 in regulation of the LPS-induced inflammatory responses of macrophages. Correlations between SUMOylation and inflammatory signaling have been widely proposed in various types of cells (18–20, 23–25, 28, 29, 31, 48–64), but studies conducted in real BMDM or RAW macrophages in innate immune contexts are scarce (18, 20, 23, 24, 28, 29). Moreover, the regulatory effects of SUMOylation on the LPS-induced inflammatory responses of macrophages remain obscure. One SUMO protease, SENP6, has been found to negatively regulate TLR inflammatory signaling of macrophages by deSUMOylation of NEMO, thus attenuating LPS-activated NF-κB signaling (18). In contrast, ubiquitin-conjugating enzyme 9 (UBC9) has recently been reported to blunt the proinflammatory response elicited by LPS in Kupffer cells *in vivo* and in RAW macrophages *in vitro* as demonstrated by silencing or overexpression experiments (20). Here, we found that SENP3 potentiates the LPS-induced inflammatory responses of macrophages. We provide two lines of evidence. First, silencing experiments using three sources of macrophages, *i.e.* RAW cells, BMDM from *Senp3*^+/−^ mice, and BMDM from *Senp3^flox/flox^ Lyz2-cre* mice, indicate a positive correlation between SENP3 and cytokine production in response to LPS, and also identified the MAPK/AP-1 signaling pathway that is positively modulated by SENP3. MKK7 binds and phosphorylates JNK and is, at least one, substrate of SENP3. Second, SENP3 is rapidly induced by LPS in the macrophage cytoplasm, thus allowing it to spatial-temporally regulate inflammatory signaling early in induction of the inflammatory responses. Importantly, this proinflammatory role of SENP3 is confirmed not only in an *in vitro* cell model but also in an *in vivo* endotoxin shock mouse model.

Interestingly, SENP3 in macrophages selectively catalyzes deSUMOylation of MKK7 and consequently increases JNK activity and specific MAPK/AP-1 signaling in the LPS-induced inflammatory responses. Unlike SENP6, which deSUMOylates NEMO and thus inhibits LPS-activated NFκB signaling (18), SENP3 confers no effect on NFκB signaling under LPS stimulation. This finding suggests that SENP family members play distinct roles through distinct substrates even in a similar inflammatory context.

The major site of MKK7 SUMOylation, Lys-18, is located at the extreme N terminus, which contains the docking domain of JNK (45, 46). Because JNK is a potent MAPK in macrophage TLR4 signaling, the enhancement of the MKK7–JNK interaction through MKK7 deSUMOylation may at least partially explain the importance of SENP3 in the LPS-induced inflammatory response. Notably, endogenous deSUMOylation of MKK7 is sustained even before LPS stimulation, as detected in TLR4 293 cells, in agreement with the high basal SENP3 protein level observed in these cells. In TLR4 293 cells with SENP3 knockdown, transient deSUMOylation of MKK7 after LPS stimulation was clearly visible. These data indicated that deSUMOylation of MKK7 in macrophages occurs during the LPS-induced inflammatory response, although SENP3 may have other substrates in this cellular response. Therefore, we identified an important enzyme and a substrate of SUMOylation in the innate immune responses.

The majority of reported SUMOylation substrates are transcription factors and other nuclear proteins (28, 32, 34–37, 48, 53, 65–71); SUMO conjugation usually leads to the transcriptional repression (28, 48, 53). For instance, the transactivation of IFN regulatory factor 8 is switched off by SUMO conjugation in macrophage activation (28). However, some cytoplasmic and membrane proteins can be regulated by reversible SUMOylation as well (47, 72). SENP3 can deSUMOylate MKK7, which is considered to localize in the cytoplasm (73). This interaction relies on the cytoplasmic SENP3 that remains at a low level under resting conditions but can be rapidly increased after LPS stimulation. It is worth further investigation for possible transcription-dependent effects of SENP3 in regulation of inflammation.

**Figure 7.**
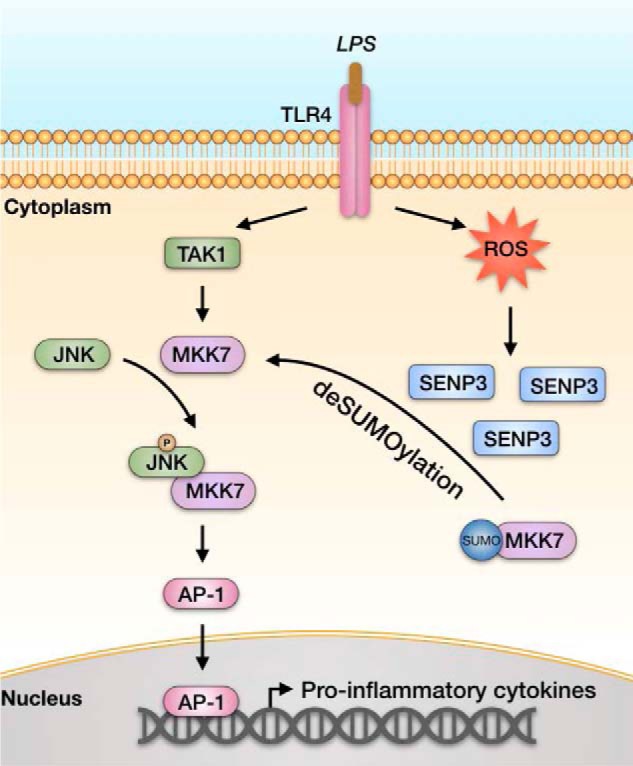
**A schematic model of SENP3 function in LPS-induced TLR4 signaling.** Under the resting condition, more MKK7 are conjugated by SUMO2/3 in macrophages. This prevents MKK7 from binding to JNK, thus attenuating the JNK phosphorylation and activation. LPS induces ROS production and consequently SENP3 accumulation in addition to activation of TLR4. SENP3 catalyzes the de-SUMOylation of MKK7 and potentiates JNK-mediated expression of inflammatory cytokines.

ROS are robustly produced from the plasma membrane and mitochondria in macrophages after cells interact with invading pathogens and bacterial components (74–78), and required for multiple LPS-triggered signaling pathways and the consequent production of cytokines, such as IL-6, TNF-α, and IL-1β (79, 80).The possibility that ROS may regulate inflammatory signaling was described in the 1990s (81). Nevertheless, the specific targets and underlying redox mechanisms have been largely unclear (82). Karin and colleagues (83) have reported that TNFα-Induced ROS cause sustained JNK activation and cell death, because of the oxidation of MAP kinase phosphatases. However, whether this type of redox regulation exists in innate immune cells has not been addressed until recently, through use of peripheral blood mononuclear cells (84). To date, the functional role of ROS has been observed under LPS stimulation contexts in the activation of the NLRP3 inflammasome, a pathway generating active caspase-1 and resulting in secretion of mature IL-1β (85–87), activation of the MAPK/AP-1 pathway and the production/secretion of IL-6 (84). The positive regulation of JNK signaling by SENP3 described in the present study is based on a prerequisite that SENP3 is rapidly induced by LPS in a ROS-dependent manner. Our previous studies have demonstrated that rapid nucleoplasmic accumulation of SENP3 is because of the oxidation of one of two cysteines in the redox-sensing domain, which leads to the blockage of ubiquitination and proteasomal degradation (33). This change in the SENP3 level and localization enables it to deSUMOylate a battery of new substrates in the nucleoplasm, thus ultimately mediating cell adaptation to oxidative stress (32–37). Therefore, we here identified a new substrate, MKK, for SENP3 in the cytoplasm and, in addition, described a novel redox mechanism for the regulation of the LPS-induced inflammatory signaling in macrophages (Fig. 7).

## Experimental procedures

### Mice and ethics statement

C57BL/6 WT mice were purchased from the Shanghai SLAC Laboratory Animal Co. Ltd. Six to 8-week-old mice were used for experiments. All mice were maintained under specific pathogen-free conditions in a barrier-sustained facility and provided with sterile food and water.

C57BL/6 *Senp3*^+/−^ mice were generated from BayGenomics (San Francisco, CA). C57BL/6 *Senp3^flox^* mice was generated by Model Animal Research Center of Nanjing University (Nanjing, China). An embryonic stem (ES) cell targeting vector containing exons 8 and 11 flanked by the loxP sequence was generated with a neomycin cassette flanked with LacZ and FRT sequence at the N terminus of exons 8-adjacent region. The vector was transfected/electroporated into ES cells. The positive ES cells were determined by PCR/Southern blot analysis and then injected into C57BL/6 mice to generate chimeric mice. Mice containing loxP, LacZ, and Neo were crossed with FLP-FRT mice to delete LacZ and neomycin. Mice containing loxP without Neo were crossed with Lyz2-cre C57BL/6 mice.

Animal experiments were carried out in strict accordance with the regulations in the Guide for the Care and Use of Laboratory Animals issued by the Ministry of Science and Technology of the People's Republic of China. The protocol was approved by the Institutional Animal Care and Use Committee of Shanghai Jiao Tong University School of Medicine (permit number: A-2016-023). All surgery was performed under sodium pentobarbital anesthesia, and every effort was made to minimize suffering.

### Cell culture and transfection

RAW264.7 and HEK293T were purchased from the American Type Culture Collection (Manassas, VA, USA); TLR4 HEK293 was kindly provided by Dr. Zizhen Kang (Shanghai Institute of Immunology, China). RAW264.7, HEK293T, and TLR4 HEK293 cells were cultured in Dulbecco's modified Eagle's medium (HyClone, Logan, UT). All media were supplemented with 10% fetal bovine serum (Gibco), 100 units/ml of penicillin, and 100 mg/liter of streptomycin (Gibco). Cells were maintained at 37 °C in a humidified atmosphere with 5% CO_2_. In HEK293T and TLR4 HEK293 cells, transfection with plasmid DNA and siRNAs was performed using Lipofectamine 2000 (Invitrogen), whereas in RAW 264.7 cells transfection was performed using Attractene Transfection Reagent (Qiagen, Germany), all following the manufacturer's instructions.

### siRNA, plasmid, and mutagenesis

The siRNA specific for SENP3 and nonspecific control siRNA oligonucleotides were synthesized and used as previously described (32). The plasmids of FLAG–SENP3, RGS.His–SENP3 (RH-SENP3 for short), GFP–SENP3, and GFP–SENP3 mutant C532A were constructed and used previously (33). The plasmid pCDNA–MKK7 was kindly provided by Dr. Guoliang Xu (Institute of Biochemistry and Cell Biology, Chinese Academy of Sciences, China). Based on pCDNA–MKK7, we generated FLAG–MKK7 and FLAG–MKK7–SUMO3 plasmids by subcloning, and FLAG–MKK7 Lys/Arg mutant by site-directed mutagenesis using a QuikChange Mutagenesis Kit (Agilent Q5 Technologies) as described (37). Based on FLAG–MKK7 and FLAG–MKK7–SUMO3, we generated GST–MKK7 and GST–MKK7–SUMO3 plasmids by subcloning. The sequences of the siRNA oligonucleotides for murine SENP3 and the primers used in the plasmid mutagenesis were as listed: si-SENP3–1, 5′-GUAAAGAGGUGGACCAAAAdTdT-3′; si-SENP3–2, 5′-GGGCUGGAAAGGUUACUUCAAdTdT-3′; K18R MKK7 forward, 5′-GTTCTCCTGCCTCAGCTTTGCTTCCAGGCG-3′; K18R MKK7 reverse, 5′-CGCCTGGAAGCAAAGCTGAGGCAGGAGAAC-3′; K400R MKK7 forward, 5′-GCGGTGACTCAGTCCTCGCCATGACATCC-3′; and K400R MKK7 reverse, 5′-GGATGTCATGGCGAGGACTGAGTCACCGC-3′.

### BMDM-derived macrophages culture

BMDM were differentiated from hematopoietic precursor cells of *Senp3*^+/−^ or *Senp3^flox/flox^ Lyz2-cre* mice. Mice were dissected and sterilized with 70% ethanol under sodium pentobarbital anesthesia. The bones were flushed with a syringe with RPMI 1640 (HyClone) to extrude bone marrow. Cell suspensions were filtered through a 40-μm cell strainer and incubated for 2 min with Red Blood Cell Lysing Buffer. The bone marrow cells were induced to differentiate in RPMI 1640 supplemented with 2 mm l-glutamine, 10% fetal bovine serum, penicillin/streptomycin, and 50 ng/ml of murine macrophage colony-stimulating factor (M-CSF) (PeProtech, Rocky Hill, NJ). Fresh murine macrophage colony-stimulating factor (final concentration, 50 ng/ml) was re-added at day 3. Media was changed at day 5 and BMDM were matured at day 7. Immunostaining of F4/80 and CD11b was used to identify the characteristics of these cells by flow cytometry. Fluorescein isothiocyanate-conjugated anti-F4/80 (11-4801-81) was purchased from eBioscience (San Diego, CA). APC-conjugated anti-CD11b (553312) was purchased from BD Bioscience (San Jose, CA).

### Quantitative RT-PCR (qRT-PCR)

This method was as previously described (37). Total RNA was isolated from cells and tissues using TRIzol reagent (Invitrogen), and cDNA was synthesis using TRIzol reagent (Invitrogen). Quantitative real-time PCR was conducted using SYBR Green (Roche, Switzerland) on the ABI Prism 7500 system. GAPDH expression was used as internal control. The primer sequences used were as follows: IL-6 forward, 5′-TGCAAGAGACTTCCATCCAG-3′; IL-6 reverse, 5′-ATTTCCACGATTTCCCAGAG-3′; TNFα forward, 5′-TCGTAGCAAACCACCAAGTG-3′; TNFα reverse, 5′-TTGTCCCTTGAAGAGAACCTG-3′; IL-1β forward, 5′-AAGAGCCCATCCTCTGTGAC-3′; IL-1β reverse, 5′-CTCATGGAGAATATCACTTGTTGG-3′; GAPDH forward, 5′-TGTGTCCGTCGTGGATCTGA-3′; and GAPDH reverse, 5′-CCTGCTTCACCACCTTCTTG-3′.

### ELISA

IL-6 and TNFα concentrations in the serum and liver were examined using mouse ELISA kits (eBioscience Systems) according to the manufacturer's instructions. For serum cytokine measurement, blood was collected from all experimental mice and allowed to clot for 2 h at room temperature before centrifugation for 30 min at 2,000 × *g*. Serum was harvested and stored at −80 °C for subsequent assays. For hepatic cytokine measurement, liver tissue was homogenized and centrifuged for 30 min at 12,000 × *g* at 4 °C. Supernatant was acquired and stored at −80 °C for subsequent assays.

### Immunoblotting (IB)

IB was performed using the routine methods as described before (32). The antibodies against SUMO2/3 (4971), SENP3 (5591), JNK (9252), phospho-SPAK/JNK (Thr-183/Tyr-185) (4668), p38 (8690), phospho-p38 (Thr-180/Tyr-182) (4511), ERK (4695), and phospho-ERK (Thr-202/Tyr-204) (4370) were purchased from Cell Signaling Technology (Beverly, MA). The antibody against IκBα (sc-371) was purchased from Santa Cruz Biotechnology (Santa Cruz, CA). The antibodies against GFP (ab290), MKK7 (ab52618), c-Jun (ab32137), and poly(ADP-ribose) polymerase (ab32138) were purchased from Abcam (Cambridge, MA). The antibody against RH (34610) was purchased from Qiagen. The antibodies against FLAG (F3165), β-actin (A5441), and tubulin (T6199) were purchased from Sigma.

### FLAG immunoprecipitation assay

The method was as previously described (37). Transfected cells were lysed in a lysis buffer (50 mm Tris-HCl, pH 7.4, 150 mm NaCl, 1 mm EDTA, and 1% Triton X-100). Anti-FLAG M2 Affinity Gel (A2220, Sigma) was added to the cell lysates and incubated overnight at 4 °C. The beads were washed four times in the lysis buffer. After the last wash, FLAG-tagged proteins were eluted in elution buffer (lysis buffer containing mixture protease inhibitor (Roche) and 20 mm *N*-ethylmaleimide (Sigma)) and then subjected to IB.

### Co-immunoprecipitation (co-IP) assay

The method was performed as previously described (37). Cells were lysed and sonicated in RIPA buffer (Thermo Scientific) at 4 °C for 30 min, then centrifuged at 13,000 × *g* at 4 °C for another 30 min. The cell lysates were pre-cleared by adding 40 μl of Protein A/G-agarose beads (IP05, Calbiochem, Temecula, CA) per 1 ml and incubating at 4 °C for 30 min. The protein A/G beads were then removed by centrifugation. Specific antibodies were mixed with the supernatants overnight at 4 °C. Protein A/G-agarose beads were added to the lysates, and the mixture was incubated under shaking for 4 h at 4 °C. *N*-Ethylmaleimide at 20 mm was included in IP buffer to ensure SUMOylation to be conserved during manipulation. The beads were washed three times, mixed with loading buffer, and examined by SDS-PAGE and IB analyses.

### Denaturing co-IP assay

Denaturing co-IP was performed to detect the SUMO conjugates of MKK7. Cells were lysed in 150 μl of the denaturing buffer (50 mmol/liter of Tris, pH 7.4, 1% SDS) for 30 min. After boiling for 10 min, cell lysates were centrifuged for 15 min at room temperature. Supernatants were mixed with IP buffer (50 mm Tris, pH 7.4, 150 mm NaCl, 1 mm EDTA, 1% Triton X-100) and incubated with anti-FLAG M2 Affinity Gel overnight at 4 °C. The proteins were separated from the beads using IB loading buffer, then the supernatants were collected for IB.

### Ni-NTA pull-down assay

Ni-nitrilotriacetic acid resin (NTA) pull-down analysis was as previously described (32). Briefly, the cells were transfected with RH-tagged plasmid. Transfected cells were lysed in a specific lysis buffer according to the manufacturers' protocols. Ni^2+^-NTA-agarose resin (Qiagen) was then added to the cell lysates and incubated with gentle agitation at 4 °C overnight. The resin was successively washed at room temperature with four different washing buffers. After the last washing, RH-tagged proteins were eluted in elution buffer and then subjected to IB.

### GST pull-down and binding assay

GST pull-down assay is an effective way to examine the direct binding of two proteins *in vitro*. GST fusion proteins were expressed in BL21 bacteria and purified by affinity chromatography using glutathione-Sepharose (17-0756-01, GE Healthcare) as instructed by the manufacturer. Briefly, GST–MKK7 and GST–MKK7–SUMO3 fusion proteins were expressed in BL21 bacteria and lysed in lysis buffer (50 mm Tris, pH 7.4, 150 mm NaCl, 1 mm EDTA, 1% Triton X-100, 0.1 mg/ml of lysozyme solution, mixture protease inhibitor). The bacteria lysis freeze/thaw cycle was repeated 10 times and incubated with glutathione-Sepharose for 2 h at 4 °C. For the binding assay, the Sepharose were washed five times with IP buffer (50 mm Tris, pH 7.4, 150 mm NaCl, 1 mm EDTA, 1% Triton X-100, 0.1 mg/ml) and incubated with 1 μg of unactivated recombinant human JNK1 (Ag21426, Proteintech, Rosemont, IL) overnight at 4 °C. The Sepharose were washed five times, mixed with loading buffer, and examined by SDS-PAGE and IB analyses.

### Immunoprecipitation kinase assay

The kinase activity of the MKK7–JNK1 complex in phosphorylation of c-Jun was detected using a JNK Activity Screening Kit (ab65784, Abcam) according to the manufactures instructions. TLR4 293 cells were transfected with FLAG–MKK7 or FLAG–MKK7–SUMO3 plasmids. After 48 h, the cells were harvested and lysed in 200 μl of ice-cold JNK Extraction Buffer for 5 min, and then the lysates were centrifuged at 13,000 × *g* at 4 °C for another 30 min. 20 μl of anti-c-Jun fusion protein beads were added to the cell lysates and incubated overnight at 4 °C. The immunoprecipitation complexes were then washed twice with wash buffer and once with kinase buffer. The activity of MKK7 was determined in a reaction at 30 °C for 30 min in 50 μl of kinase buffer containing 50 μm ATP. The reactions were terminated by the addition of SDS sample buffer, resolved by SDS-PAGE. The phosphorylated JNK1 and c-Jun were detected by immunoblot.

### Luciferase reporter assay

RAW264.7 cells were transfected with siRNA and reporter plasmids. AP-1 reporter plasmid was kindly provided by Dr. Yi-Ching Wang (National Cheng Kung University, Taiwan). Luciferase activity was assessed with a dual luciferase assay kit (Promega) in FB12 luminometer (Berthold detection system).

### ROS detection

2′,7′-Dichlorofluorescin diacetate (DCFH-DA, Sigma) was used as an ROS capturing reagent as previously described (37).

### Nuclear and cytoplasmic fractionation

The method was as previously described (37). The cells were washed with PBS before being scraped into 1 ml of PBS. After centrifugation, cell pellets were resuspended in 0.2 ml of buffer A (10 mm Hepes-KOH, pH 7.4, 10 mm KCl, 1.5 mm MgCl_2_, 0.5 mm EDTA, 0.5 mm EGTA, plus protease mixture inhibitor (Roche)) and flushed 28 times through a 23-gauge needle syringe. The released nuclei were monitored microscopically, and purified by centrifugation. The supernatant was designated as the cytoplasmic fraction and collected with a high-speed spin to clear the debris/membranes (14,000 rpm, 10 min). The nuclear pellets were washed and resuspended in 0.1 ml of buffer B (10 mm Hepes-KOH, pH 7.4, 420 mm NaCl, 2.5% glycerol, 1.5 mm MgCl_2_, 0.5 mm EDTA, 0.5 mm EGTA, 1 mm DTT) for 30 min with gentle rotation at 4 °C. At last, the supernatant was designated as the nuclear fraction and collected with a high-speed spin to clear the debris (14,000 rpm, 10 min).

### Immunofluorescence

Cell monolayers were fixed with 4% paraformaldehyde, permeablized with 0.2% Triton X-100 and blocked with 5% BSA before incubation with primary antibodies at 4 °C overnight. The antibody against p-JNK was similar to IB antibody. The second antibody was Alexa Fluor® 488 (Invitrogen). Nuclei were stained with 4′,6-diamidino-2-phenylindole. Cells were then examined under a LSM 710 fluorescent microscope (Zeiss, Germany).

### Endotoxin shock model

For endotoxicity studies, *Senp3^flox/flox^* and *Senp3* cKO mice received an intraperitoneal injection of LPS (30 mg/kg body weight) (Sigma). At 6 h after the injection, blood was collected and serum IL-6 and TNFα were examined by ELISA. Liver, lung, and spleen were prepared for homogenates at 6 h after the injection. Using the tissue homogenates, the IL-6 and TNFα mRNAs were determined by quantitative real-time RT-PCR, and the IL-6 and TNFα proteins were determined by ELISA. Mouse survival from endotoxic shock was monitored every 8 h for up to 48 h after injection of LPS.

### Statistics analysis

Student's *t* test was used to determine the significance of the differences between two or more groups of data. Survival curves were generated using the Kaplan-Meier method, and the significance of difference was calculated by the log-tank test. A value of *p* < 0.05 was considered statistically significant.

## Author contributions

Y. L., J. Yang, and J. Yi conceived and designed the experiments. Y. L. conducted the experiments, collected data, and analyzed data. K. Y. contributed to prepare mouse tissues and use software. Z. W. contributed to establish the BMDMs. X. S. helped in ELISA. Q. Z. and X. Y. provided technical assistance in *in vivo* assays. J. C. and E. Y. provided *Senp3*^+/−^ mice. X. T. provided some reagents and suggestions. Y. L., J. Yang, and J. Yi interpreted data and wrote the manuscript. J. Yi was involved in project planning and supervision. All authors reviewed the manuscript.

## Supplementary Material

Supporting Information

## Footnotes

4 Y. Lao, K. Yang, Z. Wang, X. Sun, Q. Zou, X. Yu, J. Cheng, X. Tong, E. T. H. Yeh, J. Yang, and J. Yi, unpublished data.

## Footnotes

3 The abbreviations used are:

TLR: Toll-like receptor
AP-1: activator protein-1
PTM: post-translational modification
MAPK: mitogen-activated protein kinase
LPS: lipopolysaccharide
ROS: reactive oxygen species
BMDM: bone marrow-derived macrophage
cKO: conditional knock-out
JNK: c-Jun N-terminal kinase
IL: interleukin
TNF: tumor necrosis factor
IP: immunoprecipitation
aa: amino acid
NAC: *N*-acetyl-l-cysteine
UBC9: ubiquitin-conjugating enzyme 9
qRT: quantitative RT
IB: immunoblotting
Ni-NTA: nickel-nitrilotriacetic acid
DCFH-DA: 2′,7′-dichlorofluorescin diacetate
GFP: green fluorescent protein
ES: embryonic stem.
