# Supplementary material for: DeSUMOylation of MKK7 kinase by the SUMO2/3 protease SENP3 potentiates lipopolysaccharide-induced inflammatory signaling in macrophages
Source: J Biol Chem. 2018 Jan 19;293(11):3965–80. doi: 10.1074/jbc.M117.816769 (PMC5857993; doi:10.1074/jbc.M117.816769)
Supplement: Supporting Information [file supp_293_11_3965__index.html]

DeSUMOylation of MKK7 kinase by the SUMO2/3 protease SENP3 potentiates lipopolysaccharide-induced inflammatory signaling in macrophages — DeSUMOylation of MKK7 kinase by the SUMO2/3 protease SENP3 potentiates lipopolysaccharide-induced inflammatory signaling in macrophages — SENP3 potentiates inflammatory signaling in macrophages — Supporting Information 

# DeSUMOylation of MKK7 kinase by the SUMO2/3 protease SENP3 potentiates lipopolysaccharide-induced inflammatory signaling in macrophages

## Supporting Information

- Supplemental Figures (.pdf, 816 KB) - Supplemental Figure1-3
