# Supplementary material for: DeSUMOylation of MKK7 kinase by the SUMO2/3 protease SENP3 potentiates lipopolysaccharide-induced inflammatory signaling in macrophages
Source: J Biol Chem. 2018 Jan 19;293(11):3965–80. doi: 10.1074/jbc.M117.816769 (PMC5857993; doi:10.1074/jbc.M117.816769)
Supplement: Supporting Information [file 10.1074_M117.816769_jbc.M117.816769-1.pdf]

Fig S1. The genotypes of mice and the purity of BMDM from cKO mice are validated

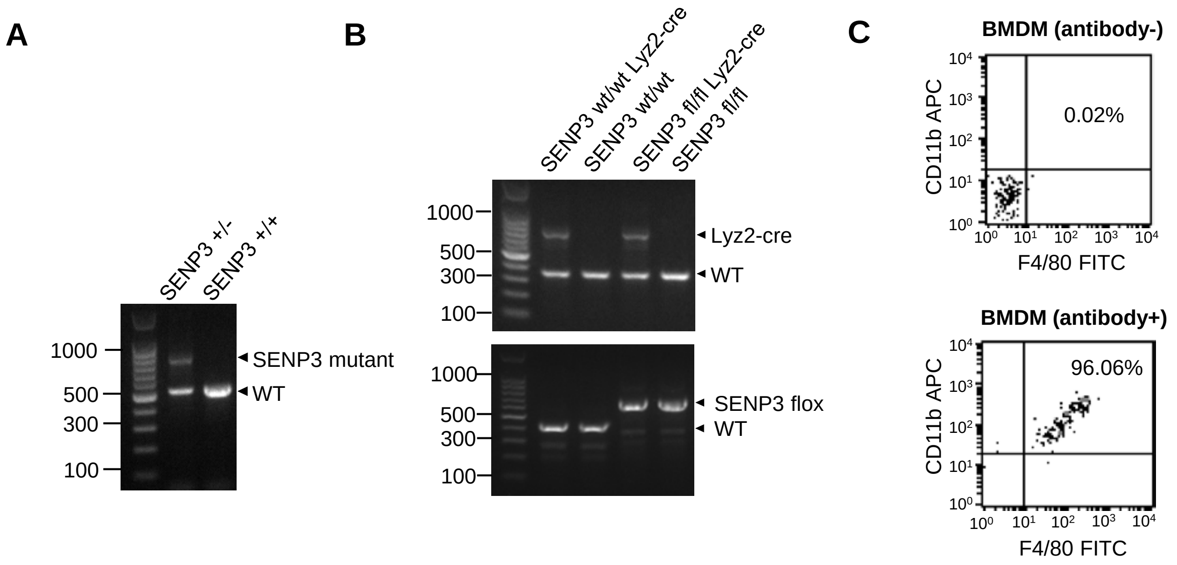

Fig S1. The genotypes of mice and the purity of BMDMs are validated.

(A) Genotyping was based on PCR primers specific for mutant SENP3 and wild type (WT) SENP3. The fragments amplified from *SENP3*$^{+/+}$ and *SENP3*$^{+/-}$ mice were shown by agarose gel electrophoresis.

(B) Genotyping was based on PCR primers specific for cre, mutant SENP3 and WT SENP3. The fragments amplified from *SENP3 fl/fl-Lyz2-cre*, WT and *SENP3 fl/wt -Lyz2-cre* mice were shown by agarose gel electrophoresis.

(C) CD11b and F4/80 flow cytometric analysis for the purity of BMDM.

Fig S2. SENP3 deficiency attenuates JNK phosphorylation

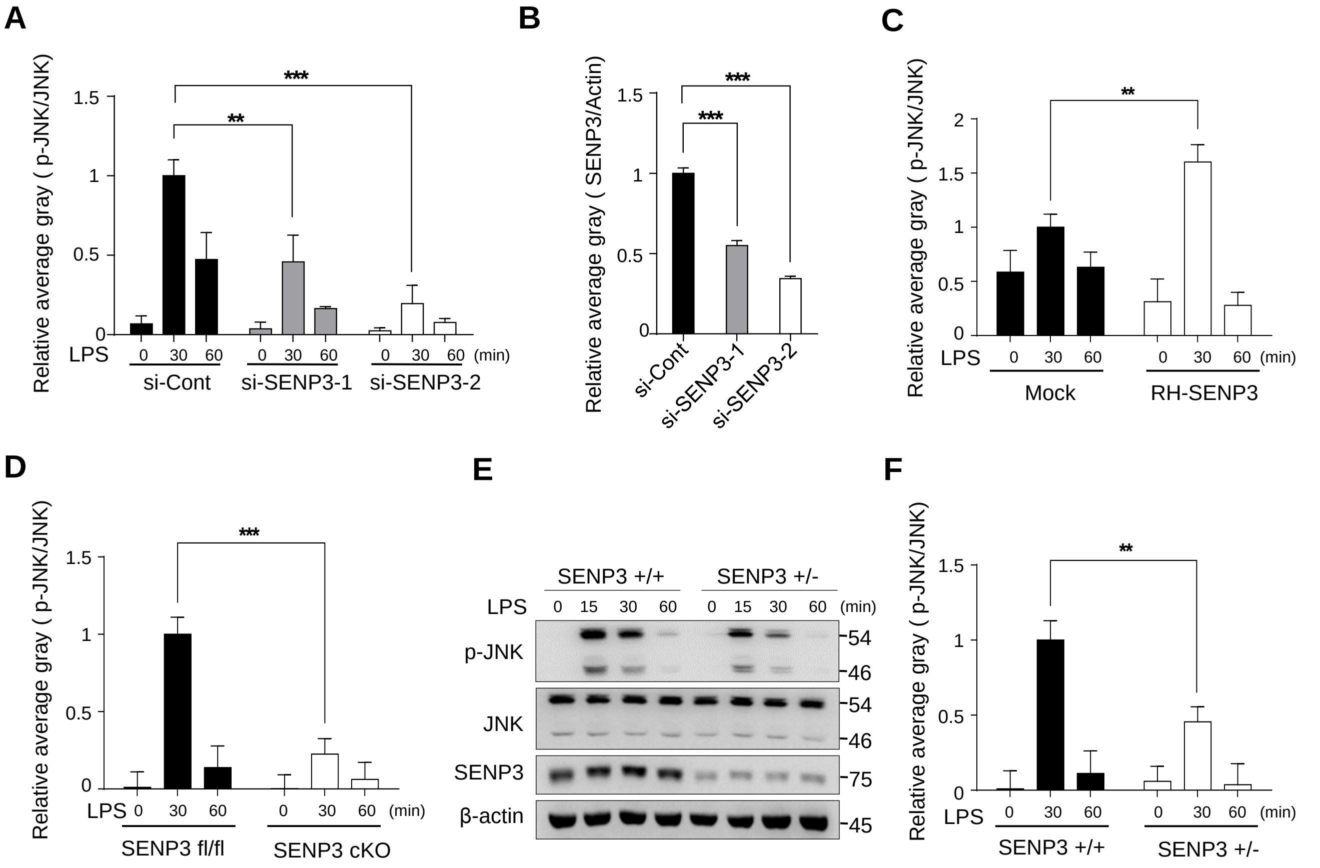

Fig S2. SENP3 deficiency attenuates JNK phosphorylation.

(A, B) Quantification of p-JNK intensity and SENP3 intensity in three independent experiments; Graphs are shown as mean ± s.d.

(C,D,F) Quantification of p-JNK intensity in three independent experiments; Graphs are shown as mean ± s.d.

(E) *SENP3*$^{+/+}$ and *SENP3*$^{+/-}$ BMDMs were stimulated with LPS (100 ng/ml) for the indicated time. p-JNK was assessed by IB.

Fig S3. The sumoylation and modification sites of MKK7 are predicted

**Protein ID:**	gi|21735544|ref|NP_660186.1|
**Defintion:**	dual specificity mitogen-activated protein kinase kinase 7 isoform 3 [Homo sapiens]
**Length:**	419 aa

```
  1 MAASSLEQKL SRLEAKLKQE NREARRRIDL NLDISPQRPR PTLQLPLAND
 51 GGSRSPSSES SPQHPTPPAR PRHMLGLPST LFTPRSMESI EIDQKLQEIM
101 KQTGYLTIGG QRYQAEINDL ENLGEMGSGT CGQVWKMRFR KTGHVIAVKQ
151 MRRSGNKEEN KRILMDLDVV LKSHDCPYIV QCFGTFITNT DVFIAMELMG
201 TCAEKLKKRM QGPIPERILG KMTVAIVKAL YYLKEKHGVI HRDVKPSNIL
251 LDERGQIKLC DFGISGRLVD SKAKTRSAGC AAYMAPERID PPDPTKPDYD
301 IRADVWSLGI SLVELATGQF PYKNCKTDFE VLTKVLQEEP PLLPGHMGFS
351 GDFQSFVKDC LTKDHRKRPK YNKLLEHSFI KRYETLEVDV ASWFKDVMAK
401 TESPRTSGVL SQPHLPFFR
```

Motifs with high probability
Motifs with low probability
Overlapping Motifs

No.	Pos.	Group	Score
1	K18	RLEAK **LKQE** NREAR	0.91
2	K400	FKDVM **AKTE** SPRTS	0.79
3	K367	LTKDH **RKRP** KYNKL	0.34
4	K236	LYYLK **EKHG** VIHRD	0.33
5	K141	WKMRF **RKTG** HVIAV	0.27

Fig S3. The sumoylation and modification sites of MKK7 are predicted. Prediction of MKK7 SUMOylation sites by software online (http://www.abgent.com/sumoplot).
